# Incidence, characteristics and risk factors of tigecycline-induced hypoglycaemia: a retrospective study from the real world

**DOI:** 10.1093/jacamr/dlaf076

**Published:** 2025-05-17

**Authors:** Bolin Zhu, Liang Liang, Di Chen, Yuanchao Zhu

**Affiliations:** Department of Pharmacy, Beijing Hospital, National Center of Gerontology, Institute of Geriatric Medicine, Chinese Academy of Medical Science, Beijing Key Laboratory of Drug Clinical Risk and Personalized Medication Evaluation, Beijing 100730, China; Department of Pharmacy, Beijing Hospital, National Center of Gerontology, Institute of Geriatric Medicine, Chinese Academy of Medical Science, Beijing Key Laboratory of Drug Clinical Risk and Personalized Medication Evaluation, Beijing 100730, China; Department of Pharmacy, Beijing Hospital, National Center of Gerontology, Institute of Geriatric Medicine, Chinese Academy of Medical Science, Beijing Key Laboratory of Drug Clinical Risk and Personalized Medication Evaluation, Beijing 100730, China; Department of Pharmacy, Beijing Hospital, National Center of Gerontology, Institute of Geriatric Medicine, Chinese Academy of Medical Science, Beijing Key Laboratory of Drug Clinical Risk and Personalized Medication Evaluation, Beijing 100730, China

## Abstract

**Objectives:**

Tigecycline is widely used in clinic because of its broad spectrum of activity including activity against drug-resistance Gram-positive and -negative microorganisms. Hypoglycaemia is a rare but potentially fatal side effect during treatment with tigecycline. At present, data on tigecycline-induced hypoglycaemia are scarce, and there is a paucity of research summarizing the clinical characteristics and incidence rate of this uncommon adverse effect of tigecycline. The purpose of this study was to assess clinical characteristics and risk factors of tigecycline-associated hypoglycaemia.

**Method:**

We performed this retrospective single-centre study of inpatients with tigecycline-induced hypoglycaemia in China between 2018 and 2024. Clinical data were achieved by review of medical records, and patients who met the inclusion criteria but did not develop hypoglycaemia were assigned as controls.

**Results:**

We finally identified 14 patients with tigecycline-induced hypoglycaemia. The incidence rate of hypoglycaemia was 1.52% (14/922) in the study population. Tigecycline-induced hypoglycaemia can happen in patients with or without diabetes and develop independent of insulin or antidiabetic drugs. For patients of tigecycline-related hypoglycaemia, intravenous dextrose was effective in the restoration of euglycemia.

**Conclusions:**

Health professional should be aware of the potential hypoglycaemia risk and monitor blood glucose level during treatment with tigecycline. For patients developing hypoglycaemia, the blood glucose monitoring should be careful and continuous even after tigecycline withdrawal.

## Introduction

Tigecycline is a glycylcycline class antibacterial drug that can exert antibacterial effects by the inhibition of bacterial protein synthesis. Its activity spectrum is broader than its congeners, such as tetracyclines. Tigecycline was approved by the FDA (Food and Drug Administration) in June 2005 and used in China in November 2011.^1^ Tigecycline has activity against resistant strains of Gram-positive bacteria, including methicillin-resistant *Saphylococcus aureus* and vancomycin-resistant *Enterococcus faecium* and *faecalis* (VRE), and Gram-negative bacteria such as *Enterobacter cloacae* and *Klebsiella pneumoniae*.^2^ For patients with renal impairment, the use of tigecycline does not require dose adjustment and has minimal drug interaction, making it suitable for elderly patients who need antibacterial therapy. Because of its obvious advantages, such as excellent antibacterial activity, wide antibacterial spectrum, good tolerance and effectiveness, tigecycline has become one of the resorts used for the treatment of multidrug-resistant bacterial infections.^3^

With the widespread use of tigecycline, an increasing number of adverse effects associated with tigecycline have been reported.^4–9^ The most common adverse effects reported for the use of tigecycline are nausea, vomiting and diarrhoea. The large randomized controlled trials showed that nausea and vomiting led to the discontinuation of tigecycline treatment in 5% patients.^5^ Furthermore, tigecycline may cause elevation in alkaline phosphatase and total bilirubin level, and coagulation disorders can also occur during tigecycline treatment.^6,7^ Recent studies have demonstrated that the use of tigecycline has a pronounced effect on fibrinogen, leading to the development of hypofibrinogenemia.^4,8,9^ Besides the previously mentioned adverse effects, hypoglycaemia is an uncommon but severe adverse effect of tigecycline treatment. Up to now, research on hypoglycaemia induced by tigecycline has been insufficient and often limited to some case reports.^10–14^ Ray *et al.* reported a non-diabetic patient suffering from tigecycline-induced severe hypoglycaemia that persisted for a prolonged period of time, and this patient had a hypoglycaemic episode with their blood glucose level down to 47 mg/dL immediately after use of tigecycline.^10^ Considering the serious condition of infected inpatients, hypoglycaemia may lead to unfavourable outcomes, such as prolonged hospitalization and increased mortality.^15^

As far as we know, present studies of tigecycline-related hypoglycaemia are limited to rare case reports, and the incidence rate of adverse reactions and the factors that may contribute to tigecycline-related hypoglycaemia have not been investigated. Thus, we implemented this retrospective single-centre study at our institution between 2018 and 2024, and a total of 971 patients treated with tigecycline during hospitalization were retrospectively analysed. Finally, 14 patients experiencing hypoglycaemia after tigecycline therapy were included in the study group. We also described the clinical characteristics of these patients, and explored the association between tigecycline exposure and the presence of hypoglycaemia. This study hopes to raise awareness among clinicians about this uncommon severe adverse effect of hypoglycaemia during treatment with tigecycline.

## Materials and methods

### Study design

This was a retrospective single-centre case series study performed in Beijing Hospital (a tertiary general hospital in Beijing, P.R. China). Data from inpatients between 1 January 2018 and 31 December 2024 were extracted from hospital information system. Patient information was recorded anonymously and used only for the purpose of study, and only researchers working on this study had access to patients’ records.

### Inclusion criteria

Patients fulfilled the inclusion criteria if: (i) patients received tigecycline treatment during hospitalization; (i) the dosages of tigecycline used were standardized based on the package inserts; and (iii) hypoglycaemia occurred after tigecycline treatment. Patients were assigned to the control group if no hypoglycaemia occurred during their hospital stay.

### Exclusion criteria

Patients were excluded from the study if they: (i) had incomplete medical records; (i) showed hypoglycaemia before tigecycline treatment; (iii) possessed potential confounding conditions that may cause hypoglycaemia such as islet cell tumour(s), hypopituitarism, adrenocortical dysfunction, liver cirrhosis, etc.; (iv) were in an abnormal physiological state that could affect blood glucose levels such as decreased food intake, increased exercise, overwork, gastrointestinal dysfunction, etc.; and (v) used other medicines that may have the potential to induce hypoglycaemia, such as fluoroquinolone.

### Study definition

Hypoglycaemia is defined as a blood glucose (BG) level <3.9 mmol/L in patients diagnosed with diabetes mellitus (DM),^16^ or <3.0 mmol/L in patients without DM.^17^

### Study analysis

All the statistical analysis was carried out using the SPSS statistics software (version 26.0, IBM). Continuous data with normal or non-normal distribution were expressed as mean ± standard deviation (mean ± SD) or median (interquartile range), and categorical data were presented using frequencies and percentages. Univariate analysis was conducted using the unpaired *t*-test for continuous variables with normal distribution, and Mann–Whitney *U*-test for continuous variables with non-normal distribution. Categorical variables were compared by *χ*^2^ or Fisher’s exact test. *P* values <0.05 were considered statistically significant.

### Ethics approval

This study, which was in compliance with the Declaration of Helsinki, received ethical approval from the Ethics Committee of Beijing Hospital (Permit Number 2022BJYYEC-312-03). Patient consent was not required because all the personal information of patients was de-identified before analysis.

## Results

### General characteristics

A total of 971 patients who received tigecycline during hospitalization were reviewed. Of these, 13 patients were excluded for the presence of hypoglycaemia before tigecycline treatment: seven patients for incomplete medical records, two patients for islet cell tumour(s), four patients for decreased food intake and nine patients for other exclusion criteria. Accordingly, the final sample comprised 936 patients who were having tigecycline therapy. Hypoglycaemia developed during tigecycline therapy in 14 patients. The prevalence of tigecycline-induced hypoglycaemia among 922 patients was 14/922 (1.52%). The average age of 14 patients was 77 years. Five patients were diagnosed with DM, and antidiabetic agents such as metformin and insulin were applied. The proportion of DM was 5/14 (35.7%) in the study group.

The demographic and clinical characteristics of 14 patients in the study group are shown in Table 1.

**Table 1.** Demographic and clinical characteristics of 14 patients with tigecycline-induced hypoglycaemia

Patient	Age/sex	DM	Antidiabetic agent	Frequency of tigecycline	Duration of tigecycline (day)	Onset after starting tigecycline (day)	Lowest BG level (mmol/L)	Number of hypoglycaemic events	Duration of hypoglycaemia (day)	Hypoglycaemia therapy	Result
P1	53/F	No	No	50 mg, q12h	14	5	2.7	Multiple	4	500 mL of 10% dextrose infusion	Improved
P2	83/F	No	No	50 mg, q12h	14	12	1.6	Twice	2	40 mL of 50% dextrose infusion	Improved
P3	75/F	No	Recombinant human insulin	50 mg, q12h	15	9	2.5	Multiple	7	10 mL of 50% dextrose infusion	Improved
P4	69/M	Yes	Metformin Insulin glargine Insulin human	50 mg, q12h	10	2	1.7	Multiple	1	40 mL of 50% dextrose infusion	Improved
P5	78/M	Yes	Insulin glargine Insulin human	50 mg, q12h	10	1	3.2	Multiple	2	20 mL of 50% dextrose infusion	Died
P6	71/F	Yes	Metformin Insulin glargine Insulin human	50 mg, q12h	4	1	2.1	Multiple	2	20 mL of 50% dextrose infusion	Improved
P7	78/M	Yes	Insulin glargine Insulin human	50 mg, q12h	9	1	3.6	Twice	5	20 mL of 50% dextrose infusion	Died
P8	71/M	Yes	Insulin glargine Insulin human	50 mg, q12h	4	2	1.8	Once	1	40 mL of 50% dextrose infusion	Died
P9	89/F	No	No	50 mg, q12h	9	2	0.7	Multiple	2	40 mL of 50% dextrose infusion	Improved
P10	91/F	No	No	50 mg, q12h	23	4	2.9	Multiple	6	40 mL of 50% dextrose infusion	Improved
**P11**	**74/M**	No	No	50 mg, q12h	9	3	2	Multiple	10	40 or 20 mL of 50% dextrose infusion	Improved
P12	81/M	No	No	100 mg, q12h (first 3 days) 50 mg, q12h (last 2 days)	5	2	2.7	Multiple	2	500 mL of 10% dextrose infusion	Improved
**P13**	**84/M**	No	No	50 mg, q12h	6	1	2.6	Multiple	5	20 mL of 50% dextrose infusion	Improved
P14	81/M	No	No	50 mg, q12h	16	6	2.4	Multiple	9	20 mL of 50% dextrose infusion	Improved

P, patient; F, female; M, male; q12h, twice a day.

### Hypoglycaemia in the 14 patients

All patients except for Patient 12 were treated with tigecycline using normal-dose treatment (50 mg, q12h). Only Patient 12 underwent high-dose treatment (100 mg, q12h for the first 3 days, and then 50 mg, q12h for the last 2 days). The mean duration of tigecycline treatment was 10.57 days. The onset of hypoglycaemia occurred within 3.64 days on average. Generally, eight patients developed hypoglycaemia within 2 days when receiving tigecycline treatment. As depicted in Table 1, the onset time of hypoglycaemia for Patient 2 was 12 days after starting tigecycline treatment, with a lowest BG level of 1.6 mmol/L. This may be because the definition of hypoglycaemia used in this study was relatively strict (BG level <3.0 mmol/L for a patient without DM). In fact, the BG level of Patient 2 had decreased from 4.7 to 3.5 mmol/L after having tigecycline treatment for 1 day.

The mean lowest BG level of 14 patients was 2.32 mmol/L, and the duration of hypoglycaemia for these patients was 3.92 days. All patients were clinically given a dextrose infusion when developing hypoglycaemia, and symptoms were promptly relieved and the BG level increased. For 14 patients who underwent tigecycline-induced hypoglycaemia, seven patients (50% in the study group) continued to use tigecycline and received glucose supply. Before tigecycline withdrawal, the hypoglycaemia of these seven patients was well controlled. For six patients in the study group, the hypoglycaemia showed improvement after the discontinuation of tigecycline. One patient (Patient 11) was a special case, as their BG level did not recover and the hypoglycaemia lasted for several days after the withdrawal of tigecycline.

### Risk factors for tigecycline–associated hypoglycaemia

The clinical characteristics of the patients included in the study group and control group have been described and compared in Table 2. The ages of the hypoglycaemia group and non-hypoglycaemia group were 77 ± 9.54 and 71.20 ± 16.84 years, which were statistically significant between two groups (*P* = 0.043). The length of hospital stay of patients in the hypoglycaemia group (40.79 ± 15.23 days) was longer than that in the non-hypoglycaemia group (29.02 ± 22.61 days), but the difference between the two groups was not significant (*P* = 0.053). In terms of patient gender, the ratio of male-to-female was 1.33:1 for the hypoglycaemia group, and 1.85:1 for the non-hypoglycaemia group. The statistical analysis showed that no significant difference in gender was observed between the two groups (*P* = 0.549). From the coexisting disorder perspective, patients who had underlying medical conditions of malnutrition (*P* = 0.015), anaemia (*P* = 0.038), viremia (*P* = 0.000), gastrointestinal haemorrhage (*P* = 0.041) and acute renal failure (*P* = 0.017) were significantly more likely to develop tigecycline-associated hypoglycaemia than the control group (Table 2). Conversely, no significant difference was detected between two groups in terms of other coexisting disorder, including hypertension (*P* = 0.620), coronary heart disease (*P* = 0.733), dyslipidaemia (*P* = 0.467), diabetes (*P* = 0.942), hypoproteinaemia (*P* = 0.352), dyspepsia (*P* = 0.254), respiratory failure (*P* = 0.209), bacteraemia (*P* = 0.105) and abnormal liver function (*P* = 0.581).

**Table 2.** Clinical characteristics of the patients at baseline

Characteristics	Patients with hypoglycaemia (*N* = 14)	Patients without hypoglycaemia (*N* = 922)	*P* value	OR (95% CI)
Age (mean ± SD (range))	77 ± 9.54 (53–91)	71.20 ± 16.84 (12–103)	**0**.**043**	—
Hospital length of stay, days (mean ± SD)	40.79 ± 15.23	29.02 ± 22.61	0.053	—
Gender, *n* (%)			0.549	1.384 (0.476–4.024)
Male	8 (57.14%)	598 (64.86%)		
Female	6 (42.86%)	324 (35.14%)		
Underlying diseases, *n* (%)				
Hypertension	9 (64.29%)	532 (57.70%)	0.620	1.320 (0.439–3.968)
Coronary heart disease	5 (35.71%)	290 (31.45%)	0.733	1.211 (0.402–3.645)
Dyslipidaemia	5 (35.71%)	249 (27.01%)	0.467	1.502 (0.498–4.524)
Diabetes	5 (35.71%)	338 (36.66%)	0.942	0.960 (0.319–2.888)
Other disorder, *n* (%)				
Malnutrition	8 (57.14%)	255 (27.66%)	**0**.**015**	3.488 (1.198–10.150)
Hypoproteinaemia	10 (71.43%)	545 (59.11%)	0.352	1.729 (0.539–5.555)
Dyspepsia	1 (7.14%)	22 (2.39%)	0.254	3.147 (0.394–25.125)
Respiratory failure	9 (64.29%)	437 (47.40%)	0.209	1.998 (0.664–6.006)
Anaemia	12 (85.71%)	536 (58.13%)	**0**.**038**	4.321 (0.962–19.416)
Viremia	2 (14.29)	13 (1.41%)	**0**.**000**	11.654 (2.367–57.372)
Bacteraemia	3 (21.43%)	82 (8.89%)	0.105	2.794 (0.764–10.216)
Gastrointestinal haemorrhage	7 (50%)	238 (25.81%)	**0**.**041**	2.874 (0.998–8.279)
Abnormal liver function	5 (35.71%)	267 (28.96%)	0.581	1.363 (0.453–4.104)
Acute renal failure	3 (21.43%)	55 (5.97%)	**0**.**017**	4.299 (1.165–15.860)

*P* values highlighted in **bold** denote statistical significance at a <0.05; ‘—’, OR was not available for unpaired t-test.

### Tigecycline treatment

Table 3 shows the tigecycline treatment details of patients in hypoglycaemia groups and non-hypoglycaemia groups. We found that the duration of tigecycline treatment of patients in the study group (10.41 ± 5.42 days) was slightly higher than that in the control group (9.41 ± 4.89 days), but the difference was not significant (*P* = 0.450). In addition, no significant difference was found between the two groups in terms of total dose and mean daily dose of tigecycline treatment.

**Table 3.** Tigecycline use in patients with and without hypoglycaemia

Tigecycline treatment details	Patients with hypoglycaemia (*N* = 14)	Patients without hypoglycaemia (*N* = 922)	*P* value
Duration of tigecycline use, days, (mean ± SD)	10.41 ± 5.42	9.41 ± 4.89	0.450
Duration of tigecycline use, days, *n*, (%)			
<7 days	4 (28.57%)	349 (37.85%)	0.477
≥7 days and <14 days	6 (42.86%)	396 (42.95%)	0.994
≥14 days	4 (28.57%)	177 (19.20%)	0.378
Total dose of tigecycline, mg, (mean ± SD)	1105.00 ± 528.75	990.41 ± 489.05	0.385
Mean daily dose of tigecycline, mg/day, (mean ± SD)	109.10 ± 12.20	107.98 ± 7.68	0.592

### Case report

#### Case 1

**Figure 1.**
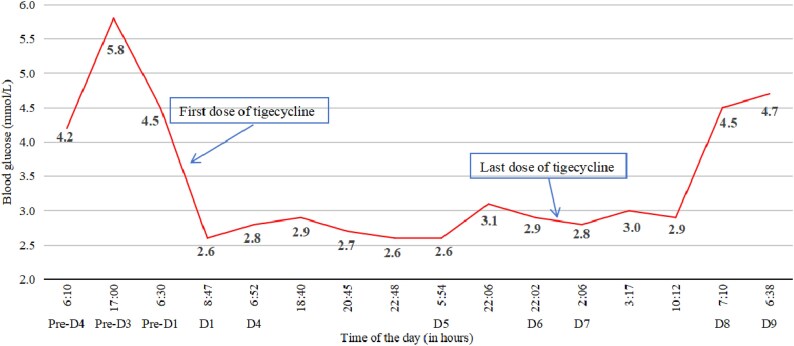
The BG levels profile of Patient 13 before and after tigecycline treatment.

Patient 13 was an 84-year-old male who was admitted to the hospital because of septic shock, along with an increased temperature of 38°C. The BG levels were within the normal range and the patient had no history of diabetes. At admission, antibiotic treatment was started with meropenem (0.5 g, q12h). On the day 2 of admission, his body temperature returned to normal, and blood examinations showed a decrease in infection markers. On day 7, the patient had a fever again, and chest CT revealed pneumonia. Then, the antibiotic regimen changed to meropenem and vancomycin. On day 9, abdominal CT showed gallstone, oedema of the gallbladder wall, swelling of the psoas major and quadratus lumbois, and thus tigecycline (50 mg, q12h) was given for complex intra-abdominal infections. On day 1 of tigecycline treatment, the patient experienced hypoglycaemia with BG level decreased to 2.6 mmol/L. The hypoglycaemia was promptly relieved with 20 mL of 50% dextrose infusion. The changes in BG levels of Patient 13 are shown in Figure 1. No hypoglycaemic event occurred within the next 3 days. On day 4 of tigecycline treatment, the patient suffered hypoglycaemia again. This time, hypoglycaemia was sustained, and three further hypoglycaemic events occurred on the same day. Each time, the BG level was restored to normal with intravenous 50% glucose. After 6 days of tigecycline treatment, infection markers in Patient 13 decreased and they did not experience fever. Tigecycline was stopped, and the hypoglycaemia was finally relieved 12 hours after the last dose of tigecycline. Starting from 1.5 days since the last dose of tigecycline treatment, the BG levels started to rise gradually and were well maintained within the normal range (Figure 1).

#### Case 2

**Figure 2.**
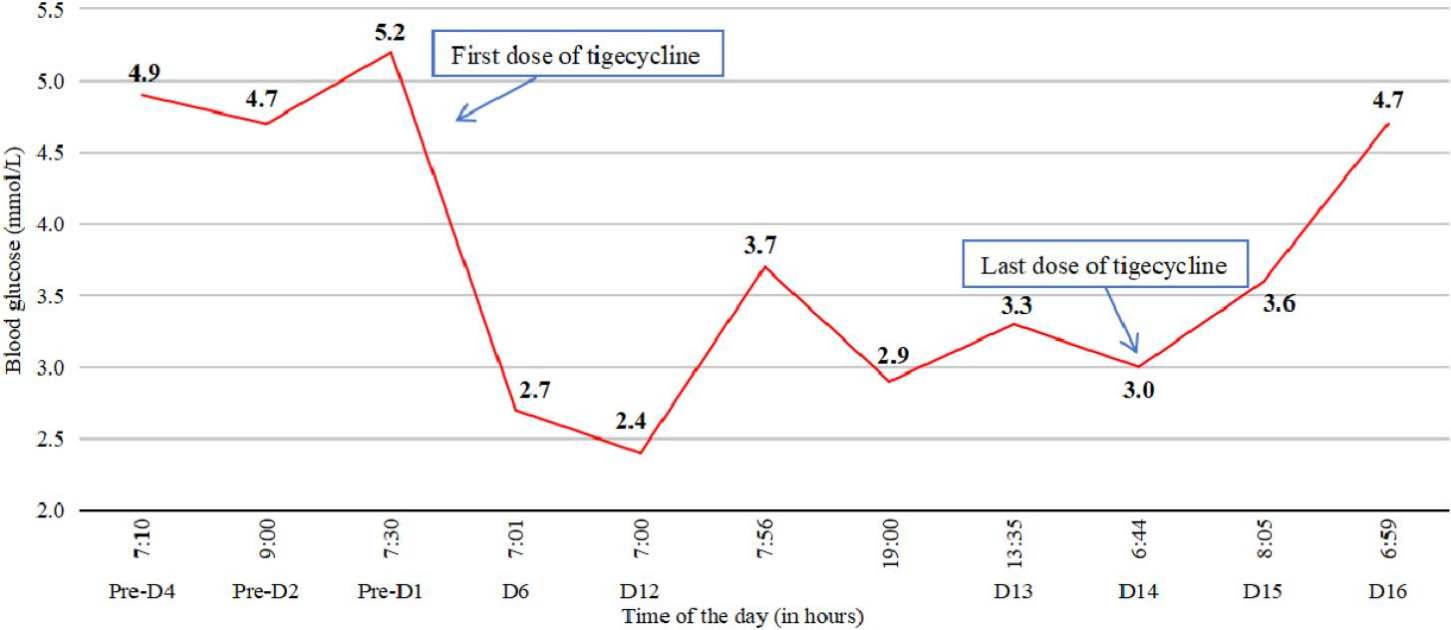
The BG levels profile of Patient 14 before and after tigecycline treatment.

Patient 14 was an 80-year-old male admitted to our hospital with pneumonia. After admission, antimicrobial susceptibility testing indicated the presence of *K. pneumoniae*, and thus ceftazidime-avibactam (2.5 g, q8h) was used. After 6 days of admission, infection had not been well controlled, and the antibiotic regimen was changed to tigecycline because of the positive of *Acinetobacter baumannii*. This patient had no history of diabetes, and the BG levels were within the normal range before tigecycline therapy. After 6 days of tigecycline therapy, the patient suffered severe hypoglycaemia with a BG level of 2.7 mmol/L. He was given 20 mL of 50% dextrose infusion, after which symptoms were relieved 15 min later and the BG increased to 6.7 mmol/L. Figure 2 shows Patient 14’s BG levels during this hospitalization. No hypoglycaemic event happened within the next 6 days. On day 12 of tigecycline therapy, the patient had a hypoglycaemic event immediately after administration of the morning dose of tigecycline, manifesting with severe palpitations, tremors, and sweating. The BG level was 2.4 mmol/L, which was managed again with dextrose infusion. Similar hypoglycaemia happened on the night of the same day. Before the withdrawal of tigecycline, hypoglycaemia happened almost every day. Tigecycline was stopped on day 14 because the course of antibiotic treatment was completed. BG levels remained low for ∼1 day after the final discontinuation of tigecycline therapy (Figure 2). The patient’s BG level gradually increased to the normal range 2 days after the last dose of tigecycline.

## Discussion

Hypoglycaemia is an uncommon adverse event of tigecycline treatment. The present study first investigated the incidence rate of tigecycline-associated hypoglycaemia in the real world and its risk factors. Findings from this study can help predict which patients are at high risk of developing hypoglycaemia, and formulate monitoring strategies to prevent and timely identify hypoglycaemia for patients treated with tigecycline. In this study, 1.52% (14/922) of patients who received tigecycline treatment developed hypoglycaemia. According to the label of tigecycline approved by FDA, tigecycline-induced hypoglycaemia developed at a rate of <2%,^18^ which was similar with the incidence rate obtained in our study.

Previous case report studies have shown that tigecycline-associated hypoglycaemia can develop in patients with or without DM and occur regardless of the use of antidiabetic drugs.^10,12^ In our study, the proportion of patients with DM in the hypoglycaemia groups (35.71%) was close to that in the non-hypoglycaemia group (36.66%), and there was no statistically significant difference (*P* = 0.942) between the two groups for the rate of patients with DM (Table 2). Thus, clinicians should be vigilant for the possibility of hypoglycaemia when patients receive tigecycline therapy, no matter whether patients have DM or not.

As seen in Table 1, the onset time of hypoglycaemia varied widely in patients. Among 14 patients in the study group, eight developed hypoglycaemia within 2 days when receiving tigecycline treatment and some experienced hypoglycaemia after several days of tigecycline exposure. Therefore, clinicians should keep vigilant for the possibility of hypoglycaemia during the whole course of tigecycline therapy.

One property of hypoglycaemia associated with tigecycline therapy was considered persistent, and hypoglycaemia may be sustained for several days after tigecycline withdrawal (Patient 11). This was attributed to the fact that tigecycline has a relatively long half-life. When tigecycline was given for multiple doses (50 mg, q12h) and achieved a steady state, its elimination half-life was ∼42 hours.^18^ For these reasons, patients with tigecycline-induced hypoglycaemia required additional BG monitoring and dextrose infusion after the discontinuation of tigecycline.

In our study, statistical analysis indicated that acute renal failure may be one risk factor for hypoglycaemia related to tigecycline (*P* = 0.017). Other studies have identified renal dysfunction as a risk factor for adverse event of tigecycline, like hypofibrinogenemia and pancreatitis.^19,20^ Tigecycline is predominantly excreted via biliary route as unchanged drugs and conjugated metabolites, and <33% of the dose experiences renal excretion.^18^ Therefore, kidney related disease may cause drug over-exposure when using tigecycline, and patients are at higher risk of hypoglycaemia. When a patient develops acute renal failure, BG levels should be closely monitored if treated with tigecycline.

Up to now, the mechanism of tigecycline-associated hypoglycaemia is still unclear. Some mechanisms have been reported, such as stimulation of insulin release and increase in liver insulin sensitivity.^10^ Further study is needed to explore the potential mechanism of hypoglycaemia during tigecycline treatment.

### Conclusion

This is the first study to explore the incidence rate and risk factors of hypoglycaemia associated with tigecycline therapy. Through the retrospective analysis of hospitalized patients in the last six years, we finally found 14 patients who developed tigecycline-associated hypoglycaemia. The statistical analysis showed that the older the elderly, the higher the risk of developing tigecycline-associated hypoglycaemia, and patients who carried a diagnosis of malnutrition, anaemia, viremia, gastrointestinal haemorrhage or acute renal failure were also at increased risk. Although the incidence rate of hypoglycaemia is low (1.52%) in this study, an awareness of tigecycline-associated hypoglycaemia and its early detection and monitoring are necessary when clinicians prescribe tigecycline, regardless of whether the patient has diabetes or not. The BG levels of patients with the previously mentioned risk factors should be monitored as soon as possible after tigecycline treatment, and for patients developing tigecycline-induced hypoglycaemia, the BG levels should be continuously monitored for several days even after the withdrawal of tigecycline.

## Data Availability

All data underlying the results are available as part of the article and no additional source data are required.
