# Correction to: Incidence, characteristics and risk factors of tigecycline-induced hypoglycaemia: a retrospective study from the real world

**DOI:** 10.1093/jacamr/dlaf111

**Published:** 2025-06-16

This is a correction to: Bolin Zhu, Liang Liang, Di Chen, Yuanchao Zhu, Incidence, characteristics and risk factors of tigecycline-induced hypoglycaemia: a retrospective study from the real world, *JAC-Antimicrobial Resistance*, Volume 7, Issue 3, June 2025, dlaf076, https://doi.org/10.1093/jacamr/dlaf076

The following changes have been made to the originally published manuscript. The statement under Funding has been emended to read: “This study was supported by funding from National High Level Hospital Clinical Research Funding (BJ-2024-201, BJ-2022-173) and Drug Safety Research Project by Adverse Drug Reactions Journal (ADR2024MS09).” instead of: “This study was supported by funding from National High Level Hospital Clinical Research Funding and Drug Safety Research Project by Adverse Drug Reactions Journal (ADR2024MS09).”

